# The Atlanta Medical Society

**Published:** 1882-08

**Authors:** 


### Societies.

### THE ATLANTA MEDICAL SOCIETY.

*Reported for the Atlanta Medical Register.*

ATLANTA, July 1, 1882.

The president, Dr. W. S. Armstrong, presiding.

Dr. James B. Baird reported a case of abscess of the abdominal walls which ruptured suddenly and discharged through the umbilicus. The lady, unmarried, had complained of tenderness throughout the umbilical region for several days, and had been slightly feverish but attached no special importance to the circumstance until the hour in which he first saw the case. In an effort to rise suddenly from the bed, upon which she was reclining, the rupture occurred. She was greatly alarmed, and fainted. She was just recovering consciousness when he arrived. His diagnosis and assurances relieved her anxiety, and with the aid of an anodyne, poultices and rest in bed for two days, she made a prompt and perfect recovery.

Dr. F. H. O'Brien referred to a case recently treated. A woman, aged twenty-three years, was seized with a chill, free hemorrhage from the nose, pain in her chest, cough, and fever—the ordinary symptoms of acute lobar pneumonia. She progressed favorably and passed through the regular stages of the disease. After convalescence was fully declared she suffered a relapse. Experienced no pain, but an effusion occurred in the pleural cavity on one side. She is doing well.

Dr. G. G. Roy related the history of a case of capillary bronchitis in a child aged two and a half years. His treatment had consisted mainly of syrup of squills, paregoric and quinine, with counter-irritation to the chest. The little patient was apparently progressing well when he saw her at seven o'clock one evening, but was called at midnight in consequence of the occurrence of a convulsion, and found both lungs congested. She died in a few minutes after his arrival in a second convulsion. He attributed the unfortunate change to a sudden fall in the temperature following a thunder storm. He reported the case mainly to urge the importance of carefully shielding such patients from external influences of this character.

Dr. Baird spoke of the value of veratrum viride and opium in the treatment of these cases. The veratrum, as in pneumonia, by regulating the heart's action, lessens the amount of blood thrown upon the inflamed structure. It is invaluable, and the opium, of course, is indispensable.

Dr. O'Brien spoke favorably of the effect of iodide of potassium.

Dr. J. G. Earnest remarked that asthenia, to a marked degree, was often present in these cases. Carbonate of ammonia and heat externally applied, are then valuable means of treatment.

Dr. W. S. Elkin stated that Dr. William Pepper, of the Pennsylvania hospital, used veratrum viride in these cases from the outset. He observed that it had been suggested that the use of this remedy bled the patient internally, or into himself, so to speak, for, by dilating the systemic blood vessels the engorged vessels of the pulmonic system were depleted by a suction process, and practically the effect of venesection was thereby secured.

Dr. E. L. Connally said that he had recently treated a case of capillary bronchitis in the person of a very stout lady. Both lungs, as usual, were affected, and the most distressing dyspnœa was a prominent symptom. He used veratrum hypodermically alternating with brandy and morphia, also administered hypodermically. He at first injected the veratrum under the skin because of the urgency of the suffocative symptoms, and the effect was so happy that the same method of administration was continued. Gave two or three drops at a dose, repeated as required and gauged the effect by the doses of brandy and morphine. When the effect of the veratrum wore off the face became congested, the eyes suffused and the lips purple. Vomiting—when it occurred — was promptly arrested by the brandy and morphine injection.

The patient recovered without any untoward symptom.

Dr. J. G. Earnest was called about two weeks ago to see a lady who had had fever for a day or two; was suffering with intense pain in the head, tongue coated and bowels constipated. Ordered a mercurial cathartic and gave chloral and chloroform for the relief of the excruciating headache. The pain continuing, he abandoned the chloral mixture and, rather against his judgment, administered morphine hypodermically, with only temporary relief. He then concluded that the pain was a neuralgia affecting the nerves of the meninges, and that the other symptoms were dependent upon it, through its influence upon a highly nervous temperament. He therefore prescribed twenty grains of quinine at one dose. In two hours the fever had abated and the headache was relieved. She took forty-five grains of quinine that day and was soon well. In another and similar case one dose of quinine of twenty grains at the outset secured prompt relief.

He thought the nerves were in a condition of hyperæsthesia, and that even a slight increase over the normal supply of blood would be sufficient to excite the pain.

Dr. O'Brien said the pain was evidently neuralgia. For such conditions he prefers the bromides and aconite; gives sixty grains of bromide of sodium and three drops of tincture of aconite root every four to six hours.

